# mCSM: predicting the effects of mutations in proteins using graph-based signatures

**DOI:** 10.1093/bioinformatics/btt691

**Published:** 2013-11-26

**Authors:** Douglas E. V. Pires, David B. Ascher, Tom L. Blundell

**Affiliations:** ^1^Department of Biochemistry, University of Cambridge, Cambridge CB2 1GA, UK and ^2^ACRF Rational Drug Discovery Centre and Biota Structural Biology Laboratory, St Vincents Institute of Medical Research, Fitzroy, VIC, 3065, Australia

## Abstract

**Motivation:** Mutations play fundamental roles in evolution by introducing diversity into genomes. Missense mutations in structural genes may become either selectively advantageous or disadvantageous to the organism by affecting protein stability and/or interfering with interactions between partners. Thus, the ability to predict the impact of mutations on protein stability and interactions is of significant value, particularly in understanding the effects of Mendelian and somatic mutations on the progression of disease. Here, we propose a novel approach to the study of missense mutations, called mCSM, which relies on graph-based signatures. These encode distance patterns between atoms and are used to represent the protein residue environment and to train predictive models. To understand the roles of mutations in disease, we have evaluated their impacts not only on protein stability but also on protein–protein and protein–nucleic acid interactions.

**Results:** We show that mCSM performs as well as or better than other methods that are used widely. The mCSM signatures were successfully used in different tasks demonstrating that the impact of a mutation can be correlated with the atomic-distance patterns surrounding an amino acid residue. We showed that mCSM can predict stability changes of a wide range of mutations occurring in the tumour suppressor protein p53, demonstrating the applicability of the proposed method in a challenging disease scenario.

**Availability and implementation:** A web server is available at http://structure.bioc.cam.ac.uk/mcsm.

**Contact:** dpires@dcc.ufmg.br; tom@cryst.bioc.cam.ac.uk

**Supplementary information:** Supplementary data are available at *Bioinformatics* online.

## 1 INTRODUCTION

### 1.1 Background

Mutations play fundamental roles in evolution by introducing diversity into genomes, most often through single nucleotide polymorphisms (SNPs). Non-synonymous single nucleotide substitutions (nsSNPs) are of particular interest, as they can disrupt function by interfering with protein stability and/or interactions with partners. Such mutations can be selectively advantageous in evolution or they may cause a change in stability often leading to malfunction and resulting in disease. Thus, predicting the impacts of mutations in proteins is of major importance to understanding function, not only of molecules and cells but also of the whole organism.

Mutagenesis studies that experimentally determine free energy differences between wild-type and mutant proteins (Fersht, 1987) produce accurate results but are usually costly and time-consuming. However, the advent of databases with experimental thermodynamic parameters for both wild-type and mutant proteins such as ProTherm and ProNIT (protein-nucleic acid) (Kumar *et al.*, 2006) and more recently the SKEMPI (Moal and Fernandez-Recio, 2012), which describes protein–protein complexes, has been helpful to the study of mutations on a larger scale. These provide an experimental basis for novel *in silico* paradigms, models and algorithms to study more extensively missense mutations and their impacts on protein stability and function.

The several different approaches used to study the impacts of mutations on protein structure and function can be broadly classified into those that seek to understand the effects of mutations from the amino acid sequence of a protein alone, and those that exploit the extensive structural information now available for many proteins. The first group includes well-established and widely used sequence-based methods such as SIFT (Ng and Henikoff, 2003) and PolyPhen (Adzhubei *et al.*, 2010). Here, we focus on the second approach that takes advantage of the protein structural information that has been accumulated on the impact of mutations within the 3D space of a natively folded protein.

Structure-based approaches, which may be categorized as machine learning methods and potential energy functions, typically attempt to predict either the direction of change in protein stability on mutation (as a classification task) or the actual free energy value (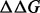) as a regression task. Machine learning-based methods have been combined with structure-based computational mutagenesis as a four-body statistical contact potential in Masso and Vaisman (2008). Support vector machines have been used to predict changes in stability from either protein sequence or structure descriptors (Capriotti *et al.*, 2005a, b; Cheng *et al.*, 2005) and more recently to predict disease-related mutations (Capriotti and Altman,2011). There have also been recent attempts to predict the stability changes on multisite mutations (Tian *et al.*, 2010). Machine learning methods have proven to be powerful predictive tools, even when data on which to train the methods have not been extensively available.

The second set of methods is based on potential energy functions. Environment-specific substitution tables, which describe the propensities of residues to mutate in a certain protein-structural environment during evolutionary time, have been used to derive a statistical potential energy function used by the method SDM (Topham *et al.*, 1997; Worth *et al.*, 2011). In the PoPMuSiC method (Dehouck *et al.*, 2009), the estimated stability change on mutation is expressed as a linear combination of 26 different energy functions, whose parameters were trained using an artificial neural network. Empirical energy functions have also been used in a method that performed Monte Carlo optimization (Bordner and Abagyan, 2004), which has also been used to study the role of conformational sampling as a way to assess the impact of single-point mutations in protein structures (Kellogg *et al.*, 2011).

Although there have been attempts to predict the affinities of particular protein–protein complexes (Moal *et al.*, 2011; Yan *et al.*, 2013), there has been much less attention to the challenge of predicting the impact of mutations on affinity in large sets of protein–protein and protein–DNA complexes. A significant exception has been the report of Guerois *et al.* (2002) on predicting the effects of mutations in a set of 82 protein–protein complexes. Another important study refers to the identification of binding energy hot spots in protein complexes by predicting the impact of mutations to alanine in >200 mutations (Kortemme and Baker, 2002). More recently, a method derived from PoPMuSiC, called BeAtMuSiC (Dehouck *et al.*, 2013), has been developed to predict the impacts of mutations on protein–protein affinities for a set of 81 proteins.

An alternative approach to study mutations is to represent residue environments as graphs where nodes are the atoms and the edges are the physicochemical interactions established among them. For instance, the method Bongo (Cheng *et al.*, 2008) attempts to predict structural effects of nsSNPs by evaluating graph theoretic metrics and identifying key residues using a vertex cover algorithm. From these graphs, distance patterns can also be extracted and summarized in a structural signature, which may then be used as evidence to train predictive models. Da Silveira *et al.* (2009) first reported the use of inter-residue distance patterns or signatures to define protein contacts, demonstrating that they are conserved across protein folds. The Cutoff Scanning Matrix (CSM) is a protein structural signature (Pires *et al.*, 2011) successfully used in large-scale protein function prediction and structural classification tasks. Pires *et al.* (2013) extended the inter-residue signature to an atomic level (aCSM) and successfully applied it in large-scale receptor-based protein ligand prediction.

Here, we use the concept of graph-based structural signatures to study and predict the impact of single-point mutations on protein stability and protein–protein and protein–nucleic acid affinity. The approach, called mutation Cutoff Scanning Matrix (henceforth called mCSM), encodes distance patterns between atoms to represent protein residue environments.

### 1.2 Method outline

The mCSM signatures are calculated in two steps:

- For a given mutation site, we define the wild-type residue environment by the atoms within a distance *r* from its geometric centre. A pairwise distance calculation between the atoms of the environment generates an atom distance matrix, which accounts for a wide spectrum of distances, from short to long range. From this matrix, distance patterns are then extracted and summarized as a feature vector;
- To account for the atom changes induced by the mutation, we introduce a ‘pharmacophore count’ vector. Each one of the 20 amino acid residues is represented by a different vector, where each position denotes the frequency of a certain pharmacophore in that residue. The difference vector between the wild-type and mutant pharmacophore vectors is then appended to the signature.

Thus, each mutation is represented as a signature vector that is used to train and test predictive machine learning methods in regression and classification tasks. In Section 2, we describe in detail how the signatures are calculated and the data sets used in this study.

### 1.3 Summary of results

We show that the mCSM signatures can be used successfully to tackle different tasks related to the prediction of the impacts of mutations in proteins. We have conducted a series of comparative experiments that indicate that mCSM performs as well as or better than several other widely used methods. mCSM is able to predict not only the direction of the change in stability of proteins and affinity of protein–protein and protein–DNA complexes but also the actual numerical experimental value, with correlation coefficients up to 0.824 for a large data set of mutations. We have also applied our methodology to predict changes of stability resulting from mutations occurring in the tumour suppressor protein p53. mCSM outperforms other methods, demonstrating its applicability to understanding mutations that lead to disease.

## 2 MATERIALS AND METHODS

### 2.1 mCSM: graph-based signatures

**Fig. 1.**
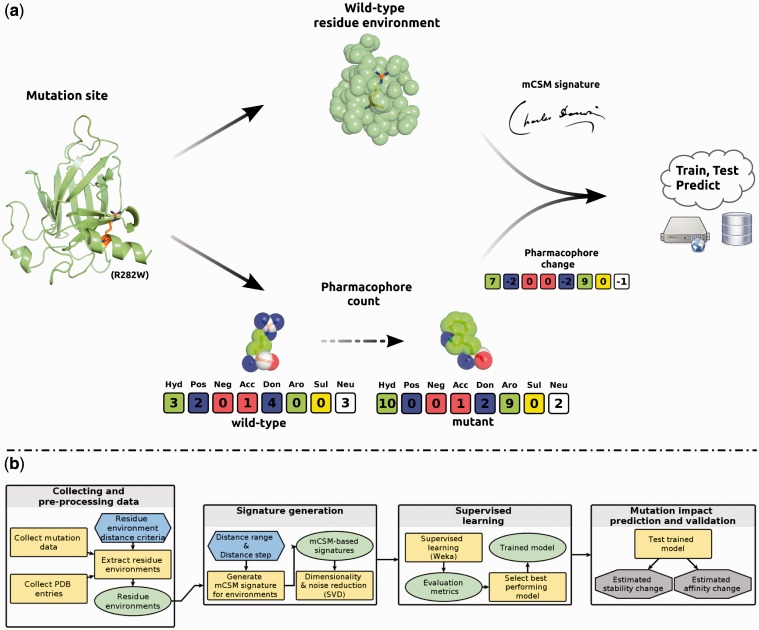
Predicting the impact of mutations with mCSM. (**a**) Highlights important steps in the methodology and how the main components of the signatures are computed. Here, we use as an example the published crystal structure of p53 (PDB ID: 2OCJ), considering the mutation site R282W, further discussed in Section 3.3. Given a mutation site in a wild-type protein, its structural environment is extracted and the distance patterns among the atoms summarized in the mCSM signature. To take into account the change in atom types due to the mutation, a pharmacophore count is performed for the wild-type and mutant residue. The changes in pharmacophore count are then appended into the signature, which is used to train/test predictive models. The considered pharmacophore types are eight: hydrophobic (green), positive (blue), negative (red), hydrogen acceptor (red), hydrogen donor (blue), aromatic (green), sulphur (yellow) and neutral (white). (**b**) Summarizes the mCSM predictive workflow that can be divided into the following steps: gathering and preprocessing the thermodynamic and structural data, extracting the residue environments, signature calculation and noise reduction, supervised learning and mutation impact prediction and validation

Here, we extend the concept of the inter-atomic distance patterns to a residue environment called mCSM. mCSM signatures can be divided into three major components:

- **Graph-based atom distance patterns:** The major components of the signatures are distance patterns in the vicinity of the wild-type residue encoded as a cumulative distribution. The wild-type residue environment, which here is defined as the set of atoms within a distance *r* from its geometric centre, can be modelled as a contact graph, where the atoms are the nodes and the edges are defined by a cutoff distance. In this way, the signatures encode distance patterns by varying the edge-defining cutoff distance, used in computing the number of edges of the resulting atomic contact graph. A cumulative distribution is obtained from an atom distance matrix, which is a pairwise distance calculation between atoms of the environment. Here, as in a previous study (Pires *et al.*, 2013), we use three types of atom classification to segment the cumulative distribution: one class (no distinction between atoms), a binary classification (atoms labelled as polar or hydrophobic) and using the Pmapper pharmacophoric classification, which classifies atoms into eight possible categories: hydrophobic, positive, negative, hydrogen acceptor, hydrogen donor, aromatic, sulphur and neutral. mCSM considers only the residue environment in the wild-type protein. In this way, our method is applicable even when no mutant structures are available. Furthermore, it does not require the generation of homology models.
- **Pharmacophore changes:** To take into account the changes in atom types due to the mutation, a ‘pharmacophore count’ vector is introduced. Wild-type and mutant residues are represented as pharmacophore frequency vectors as shown in the lower part of Figure 1a and calculated as follows. Let *L* be a set of pharmacophore types and *f* a labelling function that assigns a pharmacophore 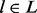 to a given atom *x*: 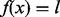. The frequency of each type of pharmacophore in a residue is then summarized in a vector *p*. The difference *p_change_* between pharmacophore count for mutant (*p_mt_*) and wild-type (*p_wt_*) residue is calculated (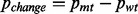) and appended to the signature. The atom pharmacophores are characteristics described by PMapper and belong to eight possible classes: hydrophobic, positive, negative, hydrogen acceptor, hydrogen donor, aromatic, sulphur and neutral.
- **Experimental conditions:** The experimental conditions, in which the thermodynamic data, such as pH and temperature are collected, are also appended to the signatures when available. Relative solvent accessibility of the residue is also included.

Figure 1b summarizes the mCSM prediction workflow as follows: preprocessing the thermodynamic and structural data, extracting the residue environments, signature calculation and noise reduction, supervised learning and mutation impact prediction and validation.

Algorithm 1 shows the function that calculates the proposed mCSM signature, which requires the following input parameters: a set of mutations, wild-type structure and the atomic categories (or pharmacophore) to be considered, a cutoff range (*D_MIN_* and *D_MAX_*) and a cutoff step (*D_STEP_*) in which each cutoff is discretized. For each mutation the residue environment is calculated based on a cutoff distance, selecting all interacting atoms in the residue vicinity. The pairwise distances between all pairs of atoms of the residue environment are then calculated and stored in a distance matrix. The distance matrix is scanned considering the cutoff range and cutoff step generating a cumulative distribution of the distances by atomic category. Finally, the pharmacophoric changes between wild-type and mutant residue are appended to the signature as well as the experimental conditions (pH and temperature) and residue relative solvent accessibility. The generated signatures are then used as evidence to train predictive classification and regression models.

**Algorithm 1: Mutation Cutoff Scanning Matrix Calculation.**

1: **function** mCSM(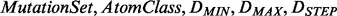)

2: **for all** mutation 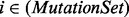 **do**

3:  *residue_environment* = extractResidueEnvironment(*mutation*)

4:  
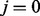

5:  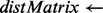 calculateAtomicPairwiseDist(*residue_environment*)

6:  **for** 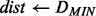; **to** *D_MAX_*; **step** *D_STEP_* **do**

7:   **for all** class 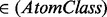 **do**

8:    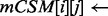 getFrequency(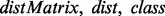)

9:    
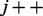

10:  add_pH_RSA_Temperature(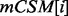)

11:  add_pharmacophores_changes(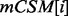)

12: **return** *mCSM*

The main goal of the mCSM signatures is to encode and concisely summarize atomic-distance patterns in the vicinity of a residue that can be correlated to the impact of a mutation. Even though interference with short-distance interactions (e.g. the creation or disruption of hydrogen bonds) is the most direct effect of mutations, mCSM signatures also take into account long-range distance patterns, in contrast with the majority of other approaches described in the literature.

It is important to note that mCSM does not define any explicit penalties, for instance when burying hydrophobic or exposing polar residues. The pharmacophore vector is used to reflect the changes in residue character and size due to the mutation, and its impact is learned without the definition of an explicit threshold. On the other hand, the perception of residue accessibility or residue depth is implicitly obtained by mCSM atomic distance patterns.

A detailed description of the evaluation methodology, supervised learning algorithms used in classification and regression tasks as well as the quality metrics used to evaluate the performance of mCSM are available as Supplementary Material.

### 2.2 Data sets

The data sets used in this work can be divided into four groups by predictive task: prediction of protein stability change on mutation, prediction of protein–protein and protein–DNA affinity change on mutation. We also describe in the Supplementary Material a dataset used to assess the ability of mCSM in predicting disease-related mutations. Table 1 summarizes the conducted experiments, the data sets and validation procedures used.

**Table 1.** Summary of data sets used, the experiments performed and validation process used

Experiment	Data set	Task	Validation	References
Protein stability change	S2648	Regression	5-fold cross-validation	(Dehouck *et al.*, 2009)
Protein stability change	S1925	Regression and classification	20-fold cross-validation	(Masso and Vaisman, 2008)
Protein stability change	S350/S309/S87	Regression	Train (S2298)	(Worth *et al.*, 2011)
Protein–nucleic acid affinity	ProNIT	Regression and classification	10-fold cross-validation	(Ahmad *et al.*, 2008)
Protein–protein affinity	SKEMPI	Regression and classification	10-fold cross-validation	(Moal and Fernandez-Recio, 2012)
Protein–protein affinity	BeAtMuSiC	Regression	10-fold cross-validation	(Dehouck *et al.*, 2013)
Disease-related mutations	KIN	Classification	20-fold cross-validation	(Capriotti and Altman, 2011)

#### 2.2.1 Protein stability change

To assess the applicability of mCSM signatures in predicting the impact of mutations in protein stability, several data sets derived from the ProTherm (Kumar *et al.*, 2006) database were considered. ProTherm is a collection of experimental thermodynamic parameters for wild-type and mutant proteins, including the change in Gibbs free energy (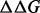). Only single-point mutations were considered. The data sets were used in comparative experiments with other methods, in regression and classification tasks, which consist of predicting the numerical value and the direction of change in 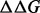, respectively.

**S2648**: The first data set, S2648, was used in comparative regression tasks where the aim is to predict the change in Gibbs free energy (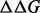) between wild-type and mutant protein. The data set comprises 2648 single-point mutations in 131 different globular proteins. For experiments with these data, we used 5-fold cross-validation, the same validation procedure use by the authors of the PoPMuSiC (Dehouck *et al.*, 2009) algorithm.

**S350**: The second data set, S350, comprised 350 mutations in 67 different proteins. It is a randomly selected subset of the S2648 data set, also used in comparative regression experiments. In this case, the remaining 2298 mutations from the S2648 data set were used to train the predictive model, whereas the S350 data set was used as a test set. This data set is widely used in the literature to compare the performance of different methods.

**S1925**: The data set S1925 was used in both regression and classification experiments. It comprises 1925 mutations in 55 proteins, which are uniformly distributed across the four major SCOP classes (Murzin *et al.*, 1995). Twenty-fold cross-validation protocol was used, the same protocol used in by the AUTOMUTE method Masso and Vaisman, 2008). 

***p53***: Finally, as a study case, we assembled a data set of 42 mutations within the DNA binding domain of the tumour suppressor protein p53, whose thermodynamic effects have previously been experimentally characterized (Ang *et al.*, 2006; Bullock *et al.* 2000; Joerger *et al.*, 2006; Nikolova *et al.* 1998, 2000). The full data set description is available as Supplementary Material.

#### 2.2.2 Protein–protein affinity change

The second set of experiments aims to assess the performance of mCSM signatures in predicting the impact of mutations on affinity of protein–protein complexes, in both regression and classification tasks (i.e. prediction of the numerical change or its direction). Affinities of protein–protein complexes were converted from molar (M) to Kcal/mol using the formulation of the Gibbs free energy (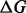):

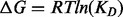

where 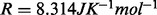 is the ideal gas constant, *T* is the temperature (in Kelvin) and *K_D_* is the affinity of the protein–protein complex. The affinity change between wild-type and mutant forms (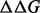) is calculated as follows:

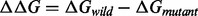



**SKEMPI**: The data set used is derived from the SKEMPI database (Moal and Fernandez-Recio, 2012). SKEMPI is a curated database that compiles changes in thermodynamic and kinetic parameters on mutation for protein–protein complexes for which a structure is available in the Protein Data Bank. From this database, which includes data for >3000 mutations, we filter only single-point mutations with available experimental affinities for both wild-type and mutant forms. This resulted in the data set that comprises 2317 mutations in 150 different proteins with available PDB structures. The SKEMPI data set used is available via Supplementary Material. Ten-fold cross-validation was used for experiments on this data set.

**BeAtMuSiC**: The data set comprises 2007 mutations used in a recent study (Dehouck *et al.*, 2013). It corresponds to a subset of the SKEMPI database, comprising mutations in 81 PDB structures. This data set was used in comparative experiments in 10-fold cross-validation as well as in a blind test, as described in Section 4 in Supplementary Material.

#### 2.2.3 Protein–nucleic acid affinity change

The third set of experiments was designed to demonstrate the capability of mCSM to predict changes in affinity in protein–DNA complexes, for both regression and classification tasks. For this purpose, we used a data set that was derived from the ProNIT database (Kumar *et al.*, 2006). ProNIT comprises experimentally determined thermodynamic interaction data between proteins and nucleic acids. In this case, we considered the change in Gibbs free energy (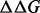). Only single-point mutations were taken into account.

**ProNIT**: The data set comprises 511 single mutations in 21 different proteins for which structures are available, and which were used in a protein–DNA interaction study that aimed to explore the relationship between free energy, sequence conservation and structural cooperativity (Ahmad *et al.*, 2008). We used 10-fold cross-validation for all experiments carried out on this data set.

## 3 RESULTS

To assess the ability of the signatures to encode the impact of mutations on protein structures, we designed an extensive series of comparative experiments with other state-of-the-art methods. In the first set of experiments, we predict the impact of single-point mutations on protein stability via regression and classification tasks. We then assess the adequacy of the signatures in predicting affinity changes on mutation at protein–protein and protein–DNA interfaces. We also perform experiments that aim to predict disease-related mutations. Finally, as a case study, we apply our methodology to predict stability changes of 42 mutations occurring in the tumour suppressor protein p53.

### 3.1 Predicting protein stability change on mutation

**Fig. 2.**
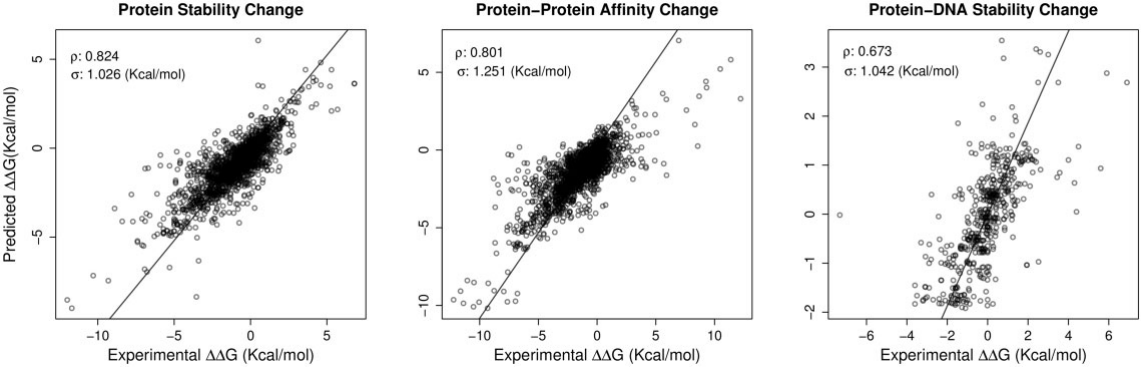
Regression results for mCSM signature predictive model trained using Gaussian processes regression for different tasks. From left to right: stability change prediction (S1925 dataset), protein–protein affinity change (SKEMPI dataset) and protein–DNA affinity change (ProNIT data set). For each data set the Pearson’s correlation coefficient (ρ) and standard error (σ) are also shown in the top-left part of each graph

As summarized in Table 1, three different stability data sets were used. The left graph of Figure 2 presents the regression results for the S1925 data set. The mCSM signatures were used to train a Gaussian process regression model that achieved a correlation of 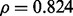 with a standard error of 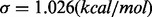. For this data set, comparing with the AUTOMUTE method (Masso and Vaisman, 2008), mCSM presented a performance equivalent or even better for both regression and classification tasks as showed in Supplementary Tables S3 and S7.

When compared with the largest available data set of mutants, S2648, used by the method PoPMuSiC (Dehouck *et al.*, 2009), mCSM presented a better performance achieving a correlation coefficient of 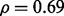 with standard error of 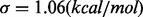, compared with a correlation of 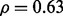 with 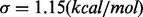 reported by the original authors. Even after 10% outlier removal, mCSM maintains its efficacy (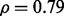 with 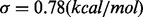 for mCSM and 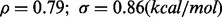 for PoPMuSiC).

The data set S350 is a subset of S2648 and was used as test set for predictive models trained with the remaining 2298 mutations. Table 2 summarizes the results obtained and shows that mCSM outperforms other approaches, some by a large margin. Because some other methods were not able to predict the stability changes for all 350 mutations, the table also shows the results for the 309 mutations for which all methods were capable of estimating a 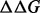 value. The performance is also shown when only mutations with 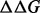 >2 kcal/mol are considered. For all cases, mCSM was the best performing method.

**Table 2.** Comparative regression experiments using the S350 data set

Method	Number of predictions	Pearson’s coefficient^a^	Standard error(kcal/mol)^a^
Automute	315	0.46/0.45/0.45	1.43/1.46/1.99
Cupsat	346	0.37/0.35/0.50	1.91/1.96/2.14
Dmutant	**350**	0.48/0.47/0.57	1.81/1.87/2.31
Eris	334	0.35/0.34/0.49	4.12/4.28/3.91
I-Mutant-2.0	346	0.29/0.27/0.27	1.65/1.69/2.39
PoPMuSiC-1.0	**350**	0.62/0.63/0.70	1.24/1.25/1.66
PoPMuSiC-2.0	**350**	0.67/0.67/0.71	1.16/1.19/1.67
SDM	**350**	0.52/0.53/0.63	1.80/1.81/2.11
**mCSM**	**350**	**0.73**/**0.74**/**0.82**	**1.08**/**1.10**/**1.48**

*Note*: Results directly obtained from Worth *et al.* (2011). Bold values highlight are the best performing metrics.

^a^The three values given per column correspond, respectively, to the whole validation set of 350 mutants, the 309 mutants for which a prediction was available for all predictors. Finally, in the third column are the results for 87 mutants, a subset of the 309 mutants, which the experimental 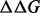 is >2 kcal/mol.

### 3.2 Predicting affinity change in protein–protein and protein–DNA complexes on mutation

The central and right graphs of Figure 2 present the regression results for the SKEMPI protein–protein data set and for the ProNIT protein–DNA data set. For the SKEMPI data set, mCSM was able to achieve a correlation of 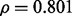 with 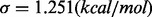, whereas for the ProNIT data set the results were 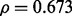 with 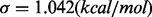. In classification tasks, for both data sets the predictive models trained with the mCSM signatures were able to achieve accuracies of >82% and Area Under ROC Curves (AUCs) of 0.826 and 0.853, respectively, as described in Supplementary Table S1.

Supplementary Table S4 shows the mCSM performance in comparison with the program BeAtMuSiC. mCSM achieves a correlation of 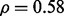 with 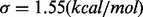, in comparison with 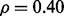 with 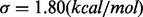 achieved by the BeAtMuSiC method. mCSM also achieves a correlation of 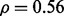 with 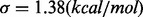 in a blind test as described in Supplementary Section S4.

We also evaluate our approach by using it to identify disease-related mutations, comparing it with well-established sequence-based methods. Supplementary Table S2 summarizes the obtained results. mCSM achieves a comparable level of accuracy, whereas presenting much better Matthews Correlation Coefficient (MCC) and AUC values. 

In addition, we performed experiments on low-redundancy data sets where all mutations in a protein (or position) are either in the test or training set exclusively, as described in Supplementary Section S4. As shown in Supplementary Tables S8 and S9, at the protein level the performance tended to be slightly inferior. The distribution of mutations per protein in the data sets is unequal, meaning that information about hundreds of mutations may be available for a single protein, which may be a significant source of bias when defining the folds in cross-validation.

### 3.3 Case study: predicting stability changes for p53 mutants

Cancer is a complex disease that arises from a combination of genetic and epigenetic changes accumulated over many years. Although there is large variability in the genes implicated in tumorigenesis, >50% of human cancers carry loss of function mutations in the transcription factor p53 (Beroud and Soussi, 2003; Olivier *et al.* 2002). In response to DNA damage, p53 transactivates a range of genes to induce cell cycle arrest, DNA repair, senescence and apoptosis, depending on the extent and types of DNA damage (Sionov *et al.*, 1999; Vousden *et al.*, 2002).

p53 is composed of three main domains: an N-terminal transactivation domain (amino acid residues 1–45), a DNA binding domain (residues 102–292) and a C-terminal oligomerization domain (residues 319–359) (Sionov *et al.*, 1999; Vousden *et al.*, 2002). Unlike most tumour suppressors that are inactivated by deletion or truncation mutations, mutations in p53 most often result in a protein with a single nucleotide substitution. The majority of these mutations (95%) are located in the DNA binding domain; they either directly interfere with residues involved in DNA binding or disrupt the wild-type conformation and stability of p53 (Olivier *et al.*, 2002). Both of these classes of mutations prevent the transcriptional activation of p53 target genes in a dominant-negative fashion (Vousden *et al.*, 2002).

A new approach in cancer therapy is to find drugs that can rescue the activity of mutant p53. This has primarily focused on trying to enhance the stability p53, potentially reducing the effect of destabilizing mutations and restoring wild-type activity. Several approaches have identified stabilizing molecules that also show a stimulatory effect on p53 DNA binding. These have included antibodies (Hupp *et al.*, 1995), peptides (Selivanova *et al.*, 1999) and small molecules identified via structure-guided design (Boeckler *et al.*, 2008) and screening approaches (Bykov *et al.*, 2002). The most advanced of these is the small molecule PRIMA-1MET (APR-246) that has successfully completed Phase I/II clinical trials, where it was observed that it could induce p53-dependent biological effects in tumour cells *in vivo* (Lehmann *et al.*, 2012).

We have used mCSM to predict the effect of mutations on the stability of p53. We used the published crystal structure of p53 (PDB ID: 2OCJ), and predicted the change in stability of >42 single mutations within the DNA binding domain of p53 whose thermodynamic effects have previously been experimentally characterized. None of these mutations was present in the training set. In addition to mCSM, we also used SDM and PoPMuSiC to predict the stability changes of these mutations. These predictions were compared directly with the experimentally determined thermodynamic effects (Supplementary Table S6).

mCSM predicted stability changes correlated strongly with the experimentally observed thermodynamic effects (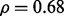), as shown in Supplementary Table S5. In addition, mCSM was a much better predictor of stability changes in p53 than either SDM (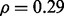) or PoPMuSiC (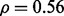), consistent with our larger analysis. In Supplementary Figure S1, we can see that compared with the experimental observations, mCSM predictions did not have a large variation. Significantly, the interquartile range and 95% confidence interval from mCSM predictions were tighter than either SDM or PoPMuSiC. 

In general there was good agreement between the algorithms in predicting the direction of change when compared with the experimental data. One interesting deviation, however, was the mutation of arginine 282 to tryptophan, clinically a commonly observed p53 mutation. Arginine 282, shown in Figure 1a and Supplementary Figure S2, is involved in a network of interactions underpinning the loop-sheet-helix major groove DNA binding motif. Mutation to tryptophan results in large structural perturbations, resulting in p53 being largely unfolded, and hence inactive, under physiological conditions (Bullock *et al.*, 2000). mCSM was able to predict accurately the effect of this mutation on the overall stability of p53. Interestingly, both SDM and PoPMuSiC predicted that the R282W mutation would be stabilizing, with SDM predicting that it would actually be stabilizing (Supplementary Table S1). In the case of SDM, the version used in the comparison considers only side chain H-bonds to main chain residues (Worth *et al.*, 2011), which tend to be less critical; however in this case the arginine side chain makes three hdydrogen bonds to other buried or partially buried side chains. This highlights the power of mCSM using the local environment to predict the effects of mutations.

In summary, we have shown that mCSM can predict the effects of mutations on the stability of p53, and can identify disease-associated destabilizing mutations. mCSM provides a reliable way to quickly assess the impact of mutations within the p53 gene, and hence the likelihood of success of stabilizing molecules, which is important within a clinical setting.

## 4 DISCUSSION AND CONCLUSIONS

We present a new approach, mCSM, for studying the impact of missense mutations in proteins. mCSM, which relies on graph-based signatures, was successfully applied and evaluated in different predictive tasks and was shown to outperform earlier methods. We have successfully applied this methodology to predict stability changes of mutations occurring in p53, demonstrating the applicability of mCSM in a challenging disease scenario.

The results achieved by mCSM support the idea that the impact of a mutation can be correlated with the atomic distance patterns surrounding an amino acid residue. The distant patterns describe the nature of the environment of the residue in the wild-type protein. They contrast with amino acid substitution patterns as used in SDM, which define the environment as a function of the residues immediately surrounding the residue. Thus, a solvent inaccessible residue in SDM will have the same defined environment whether it is on the core of the protein or close to the surface. On the other hand, the distance matrix of mCSM will differ according to the depth of the residue and the curvature of the protein surface that lies within a large radius, in this work, up to 10 Å. This is reminiscent of the focus on ‘depth’ championed by Chakravarty and Varadarajan (1999). The mCSM distance matrix will also be sensitive to the nature of the electrostatic environment, which appears to be less well described in PoPMuSiC. These advantages are evidenced in the feature selection analysis (Supplementary Fig. S3), which shows that long-range distances are the most discriminative attributes of the signatures, usually polar–polar and hydrophobic–hydrophobic atom frequencies for distances beyond 6 Å for the SKEMPI data set. A latent semantic analysis (Supplementary Fig. S4) shows that the majority of the variability of the signatures can be explained with atom frequencies for long-range distances. In this way, mCSM perceives residue environment density and depth implicitly, without relying on direct calculations or thresholds. A similar analysis was done for the other data sets (Supplementary Figs. S5 and S6).

The mCSM approach shows that a good description of the effect of mutations on the wild-type structure can be achieved using the pharmacophore count. This contrasts with use of the immediate environment of the residue to define a pharmacophore, which is de-emphasized in mCSM. Although in mCSM the pharmacophore is not described in terms of a defined structure of the mutant protein, there may still be further gains in the quality of the prediction if this could be incorporated into mCSM. A further difference from many previous methods is the omission of an assessment of the effect of the mutation on the unfolded state. This is explicitly described in perturbation methods and in programs like SDM. It appears this is either less important to the estimation of the change of stability that arises from the majority of mutations or the unfolded state is insufficiently well described in methods such as SDM. 

mCSM does not depend on the observation of mutations that have occurred in evolution of proteins. These have generally been selected over long evolutionary times for minor positive or negative changes on stability that were selectively advantageous to the organism. Mutations that occur in cancer, such as those in p53 described here, are often deleterious to the function of the protein and may not be sampled in a statistically significant manner in evolution when information on residue structural environment is required in the calculation. Therefore, they may not be properly accounted for in SDM environment-specific substitution tables. This is almost certainly the case for the mutation of arginine 282 to tryptophan in p53, which has significant effects on the stability of the protein. This underlines the importance of understanding theoretically the effects of various parameters that influence stability a major focus of both mCSM and PoPMuSiC.

In future works we intend to apply our methodology for predicting affinity changes to protein-ligand complexes, to understand the affects of mutations that occur in drug resistance in cancer and other diseases. We believe our machine learning approach is complementary to those based on potential energy functions like SDM and PoPMuSiC. This way, we intend to combine them into a hybrid method. 

## Supplementary Material

Supplementary Data
