# Supplementary material for: mCSM: predicting the effects of mutations in proteins using graph-based signatures
Source: Bioinformatics. 2013 Nov 26;30(3):335–42. doi: 10.1093/bioinformatics/btt691 (PMC3904523; doi:10.1093/bioinformatics/btt691)
Supplement: Supplementary Data [file supp_30_3_335__index.html]

mCSM: predicting the effects of mutations in proteins using graph-based signatures — mCSM: predicting the effects of mutations in proteins using graph-based signatures — mCSM: predicting the effects of mutations in proteins using graph-based signatures — Supplementary Data 

# mCSM: predicting the effects of mutations in proteins using graph-based signatures

## Supplementary Data

files

**Files in this Data Supplement:**

- Supplementary Data - pdf file
