# Supplementary material for: mCSM: predicting the effects of mutations in proteins using graph-based signatures
Source: Bioinformatics. 2013 Nov 26;30(3):335–42. doi: 10.1093/bioinformatics/btt691 (PMC3904523; doi:10.1093/bioinformatics/btt691)
Supplement: Supplementary Data [file supp_btt691_suppl.pdf]

SUPPLEMENTARY MATERIAL

1 DATA SET: DISEASE-RELATED MUTATIONS

As a complementary experiment, we assess mCSM performance in predicting disease-related mutations as a classification task. This experiment also aims to compare mCSM with well-established sequence-based predictors like SIFT (Ng and Henikoff, 2003) and PolyPhen (Adzhubei *et al.*, 2010).

KIN: the KIN data set was obtained from (Capriotti and Altman, 2011). It comprises 492 mutations occurring in 74 protein structures annotated with the Gene Ontology term "Kinase activity" (GO:0016301) and labelled either as disease-related or as neutral polymorphisms. 20-fold cross validation was used, the same procedure adopted by the original investigators who used this data set.

2 TABLES

2.1 Classification tasks

The following tables summarize the mCSM performance on classification tasks. Two possible classes of mutations were considered for the predictive tasks. Mutations were labelled as *increasing / decreasing* stability (or affinity for complexes) or, in the case of the KIN data set, *disease-related / neutral polymorphisms*.

Table 1. Classification results for the SKEMPI and ProNIT data sets considering two classes of mutations: those that increase affinity of the protein-protein or protein-DNA complex (+) and those that decrease affinity (-). 10-fold cross validation was employed for experiments on theses data sets.

Dataset	Accuracy	Precision[+]	Recall[+]	Precision[-]	Recall[-]	MCC	AUC
SKEMPI	0.828	0.687	0.436	0.851	0.942	0.451	0.826
ProNIT	0.829	0.842	0.810	0.817	0.848	0.659	0.853

Results obtained using a Random Forest classifier.

Table 2. Prediction performance for the disease-related mutations data set (KIN) considering two classes of mutations: those that are disease-related (D) and those that are considered neutral polymorphisms (P). 20-fold cross validation was used on experiments in this data set.

Method	Accuracy	Precision[D]	Recall[D]	Precision[P]	Recall[P]	MCC	AUC
SIFT	0.80	**0.90**	0.84	0.58	0.69	0.50	0.81
PolyPhen2	0.80	0.88	0.86	0.60	0.63	0.48	0.81
SVM-SEQ	0.81	0.87	0.88	0.63	0.62	0.50	0.82
SVM-3D	**0.83**	0.87	**0.91**	**0.69**	0.59	0.53	0.83
**mCSM**	**0.83**	**0.90**	0.87	0.65	**0.72**	**0.57**	**0.84**

Performance statistics directly obtained from (Capriotti and Altman, 2011). The results for the mCSM signature were generated using a Naïve Bayes classifier.

Table 3. Classification results for the S1925 data set considering two classes of mutations: those that increase stability of the protein in comparison with the wild-type form (+) and those that decrease stability (-). 20-fold cross validation was employed.

Method	Accuracy	Recall[+]	Precision[+]	Recall[-]	Precision[-]	MCC	AUC
AUTOMUTE (RF)	**0.86**	**0.70**	0.81	0.93	**0.88**	**0.66**	**0.91**
AUTOMUTE (RF-EC scores)	0.82	0.61	0.75	0.91	0.84	0.55	- - -[1]
AUTOMUTE (SVM)	0.84	**0.70**	0.75	0.90	0.87	0.61	0.86
I-Mutant 2.0	0.80	0.56	0.73	0.91	0.83	0.51	- - -[1]
**mCSM**	**0.86**	0.67	**0.82**	**0.94**	0.87	0.65	0.90

Performance statistics directly obtained from (Masso and Vaisman, 2008). [1] Metric not reported by the authors. The results for the mCSM signature were generated using a Random Forest classifier.

2.2 Regression tasks

The following tables summarize the mCSM performance on regression tasks, where the aim is to predict the actual experimental value of stability or affinity change upon mutation ($\Delta\Delta G$), which is given in *Kcal/mol*.

Table 4. Comparative prediction performance of protein-protein affinity change for the BeAtMuSiC data set. The results were obtained with low-redundancy 10-fold cross validation (at position level, as explained in Section 4).

Method	Data set	Pearson's coefficient*	Standard error(kcal/mol)*
BeAtMuSiC	S2007	0.40/0.68	1.80/1.19
**mCSM**	S2007	**0.58/0.71**	**1.55/1.00**

* The two values given per column correspond respectively to the results for whole validation set and after excluding 10% of outliers.

Table 5. Comparative prediction performance of stability change for p53 mutations. mCSM is compared to two other well established methods: SDM and PoPMuSiC.

Method	Pearson's coefficient	Standard error (kcal/mol)
SDM	0.291	1.755
PoPMuSiC	0.559	1.521
**mCSM**	**0.675**	**1.403**

Table 6. Prediction of stability changes of 42 mutations occurring on the tumour suppressor protein p53. Results for mCSM, SDM (Worth *et al.*, 2011) and PoPMuSiC (Dehouck *et al.*, 2009) methods are shown.

		Predicted ΔΔG			Absolute error		
**Mutation**	**ΔΔG**	**mCSM**	**SDM**	**PoPMuSiC**	**mCSM**	**SDM**	**PoPMuSiC**
Q104H	0.24	-1.002	0.46	0.13	1.242	0.22	**0.11**
Q104P	0.11	-0.374	-1.55	0.43	0.484	1.66	**0.32**
T123A	-0.13	-0.878	0.31	-1.09	0.748	**0.44**	0.96
A129D	-0.7	-0.700	-0.18	-0.28	**0.000**	0.52	0.42
A129E	-0.38	-0.817	-0.11	-0.20	0.437	0.27	**0.18**
A129S	-0.19	-0.819	-0.15	-0.01	0.629	**0.04**	0.18
M133L	0.30	0.198	-0.13	-0.62	**0.102**	0.43	0.92
F134L	-4.78	-3.081	-0.55	-1.60	**1.699**	4.23	3.18
V143A	-3.5	-1.917	-2.73	-3.11	1.583	0.77	**0.39**
L145Q	-2.98	-2.761	-2.11	-3.68	**0.219**	0.87	0.70
D148E	-0.43	-0.22	0.04	-0.14	**0.210**	0.47	0.29
D148S	0.22	0.219	-0.35	-0.08	**0.001**	0.57	0.30
T150P	-0.08	-0.362	0.91	0.76	**0.282**	0.99	0.84
P151S	-4.49	-2.482	-1.92	-1.90	**2.008**	2.57	2.59
V157F	-3.88	-1.328	-1.05	-1.19	**2.552**	2.83	2.69
Q165K	-1.27	-0.814	-1.18	-0.63	0.456	**0.09**	0.64
Q167E	-0.43	-0.125	0.07	0.34	**0.305**	0.50	0.77
H168R	-2.75	-1.132	-0.46	-1.14	1.618	2.29	**1.61**
R174K	-0.22	-0.686	-1.25	-0.69	**0.466**	1.03	0.47
R175A	-0.73	-0.808	-0.74	-1.12	0.078	**0.01**	0.39
R175H	-3.52	-1.041	0.26	-0.36	**2.479**	3.78	3.16
C182S	0.16	-1.079	-0.97	-0.44	1.239	1.13	**0.60**
I195T	-4.12	-2.889	-4.34	-1.95	1.231	**0.22**	2.17
L201P	0.35	-0.308	-0.71	0.72	0.658	1.06	**0.37**
V203A	0.49	-1.626	-1.01	-1.87	2.116	**1.50**	2.36
L206S	-0.1	-1.173	-1.47	-0.92	1.073	1.37	**0.82**
Y220C	-3.98	-1.897	0.48	-2.35	2.083	4.46	**1.63**
D228E	0.05	-0.266	0.01	-0.33	0.316	**0.04**	0.38
I232T	-3.19	-2.365	-3.61	-2.22	0.825	**0.42**	0.97
Y236F	0.27	-1.611	1.01	-0.94	1.881	**0.74**	1.21
M237I	-3.18	-1.839	-1.33	-0.58	**1.341**	1.85	2.60
N239Y	1.49	-0.258	1.91	-0.63	1.748	**0.42**	2.12
C242S	-3.07	-0.956	-1.06	-1.34	2.114	2.01	**1.73**
G245S	-1.21	-1.247	-0.06	-0.95	**0.037**	1.15	0.26
R248Q	-1.87	-0.166	-1.43	0.03	1.704	**0.44**	1.90
R249S	-1.92	-1.667	-1.06	-2.40	**0.253**	0.86	0.48
I255F	-3.29	-1.570	-0.76	-0.98	**1.720**	2.53	2.31
S260P	-0.32	-0.31	1.09	0.02	**0.010**	1.41	0.34
N268D	1.21	-1.609	-1.36	-1.12	2.819	2.57	**2.33**
F270C	-4.54	-1.888	0.43	-2.11	2.652	4.97	**2.43**
R273H	-0.45	-1.865	-0.47	-0.43	1.415	**0.02**	**0.02**
R282W	-3.3	-1.219	3.50	0.20	**2.081**	6.80	3.50

The first column presents the mutation (wild-type residue, position and mutant residue). The second column shows the experimental $\Delta\Delta G$ value. The next three columns present the predicted values for mCSM, SDM and PoPMuSiC, respectively. The last three columns show the absolute error in the prediction of each method.

Table 7. Regression results for the S1925 data set. 20-fold cross validation was employed.

**Method**	**Pearson's coeff.**	**Standard error(Kcal/mol)**
AUTOMUTE (REPTree)	0.79	1.1
AUTOMUTE (SVMreg)	0.76	1.2
I-Mutant 2.0	0.71	1.3
**mCSM**	**0.82**	**1.0**

Performance statistics directly obtained from (Masso and Vaisman, 2008). The results for the mCSM signature were generated using Gaussian Process regression.

3 FIGURES

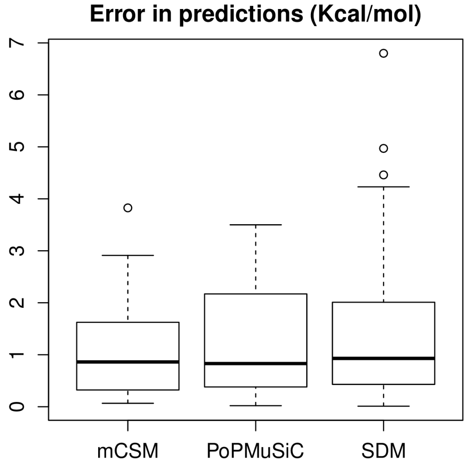

Fig. 1. Absolute error for predictions of stability change for p53 mutations for the three considered methods. A boxplot shows the absolute error distribution for the 42 p53 mutations. Even though the medians for all methods are very similar, mCSM presented a much tighter interquartile range.

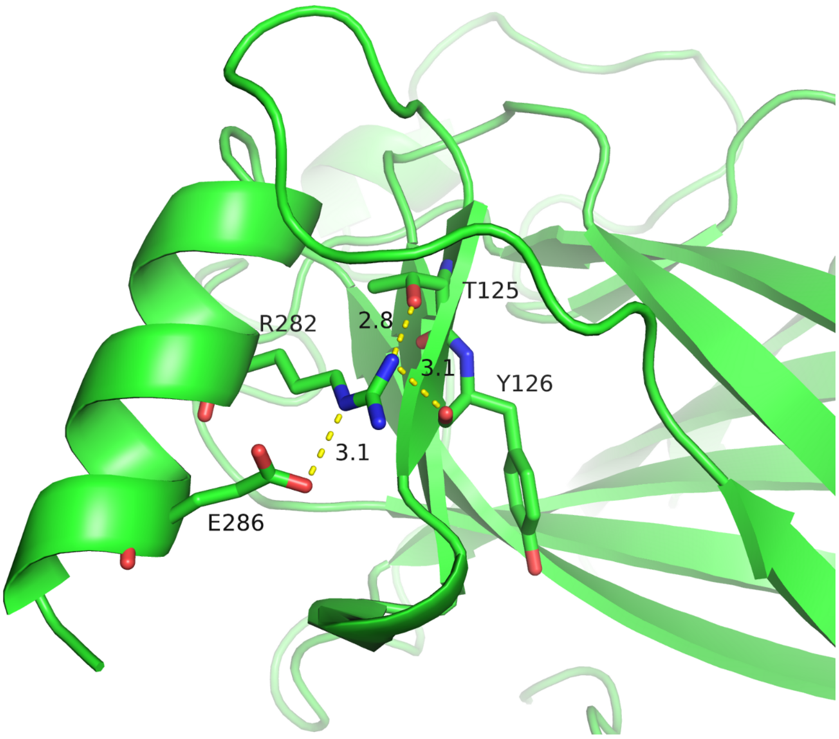

Fig. 2. Commonly observed mutation on the tumour suppressor protein p53 (arginine 282 to tryptophan). Arginine 282 is involved in a network of interactions underpinning the loop-sheet-helix major groove DNA binding motif. Mutation to tryptophan results in large structural perturbations, resulting in p53 being largely unfolded, and hence inactive, under physiological conditions (Bullock *et al.*, 2000).

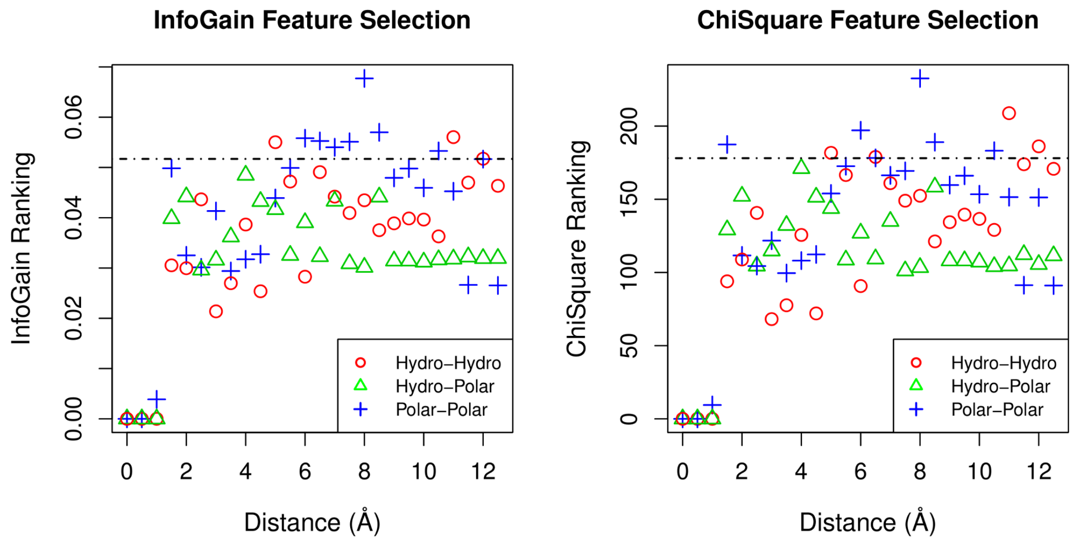

Fig. 3. Feature selection analysis for signatures generated for the SKEMPI data set (protein-protein affinity change upon mutation). The graphs show the attribute ranking according to two different feature selection approaches, which evaluate the discriminative ability of the attributes according to an Information Gain or a Chi-Square statistic (Yang and Pedersen, 1997). The signatures were generated considering two atom classes (hydrophobic and polar) for distances up to 12.5Å. The dashed lines separate the ten best-ranked attributes for each feature selection method. The most discriminative attributes seem to be long-range distance patterns, usually polar-polar and hydrophobic-hydrophobic atom frequencies for distances beyond 6Å.

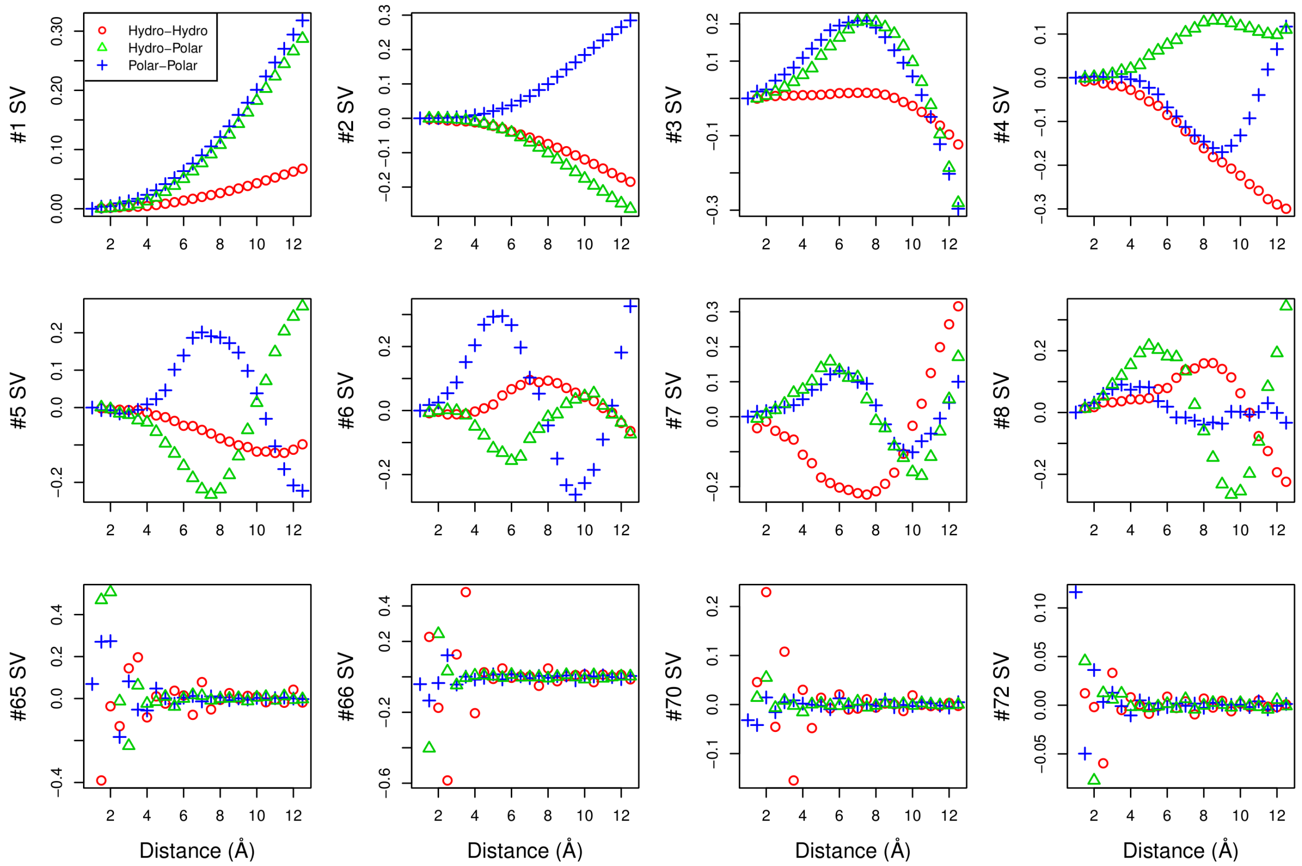

Fig. 4. Relation between original attributes and the latent variables computed by the Singular Value Decomposition for the SKEMPI data set. The signatures were generated considering two atom classes (hydrophobic and polar) for distances up to 12.5Å. The top two rows show the results for the first eight singular values. The first singular value, which account for the majority of the variability of the data, seems to be proportionally related to the number of atoms in the environment. This variability is expected since residue accessibility to solvent is variable, meaning that the environment of a core residue has a higher density than that of an exposed one. This way, mCSM perceives this density implicitly, without relying on accessibility calculations or thresholds. For the first five singular values, the long-range distance patterns seem to be dominant. On the other hand, short-range patterns are also computed, as shown in the last row of graphs.

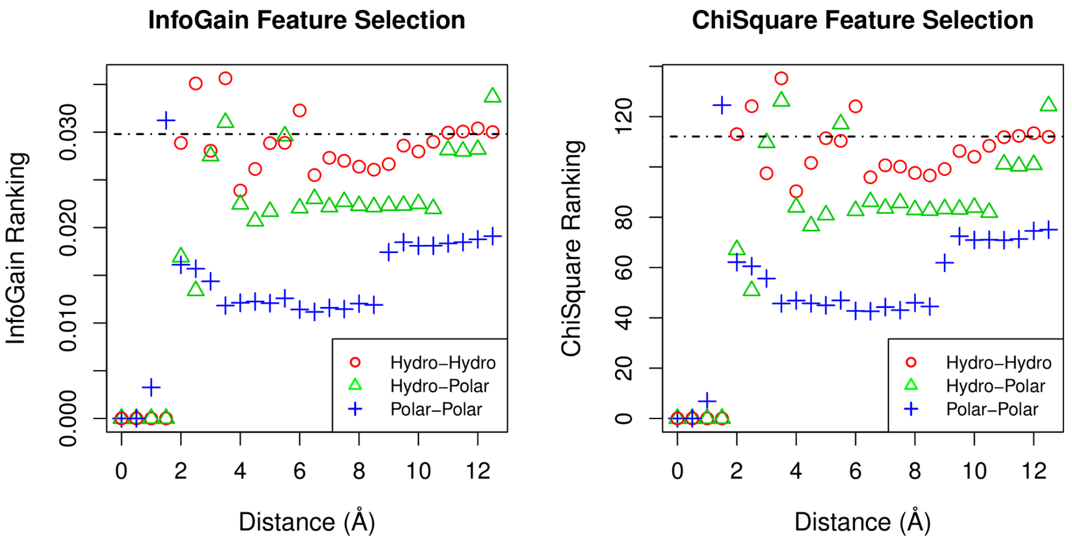

Fig. 5. Feature selection analysis for signatures generated for the S2648 data set (protein stability change upon mutation). The graphs show the attribute ranking according to two different feature selection approaches, which evaluate the discriminative ability of the attributes according to an Information Gain or a Chi-Square statistic (Yang and Pedersen, 1997). The signatures were generated considering two atom classes (hydrophobic and polar) for distances up to 12.5Å. The dashed lines separate the ten best-ranked attributes for each feature selection method. The most discriminative attributes seem to be hydrophobic-hydrophobic and hydrophobic-polar distance patterns for distances around 4Å. On the other hand, the least discriminative features seem to be polar-polar atom frequencies.

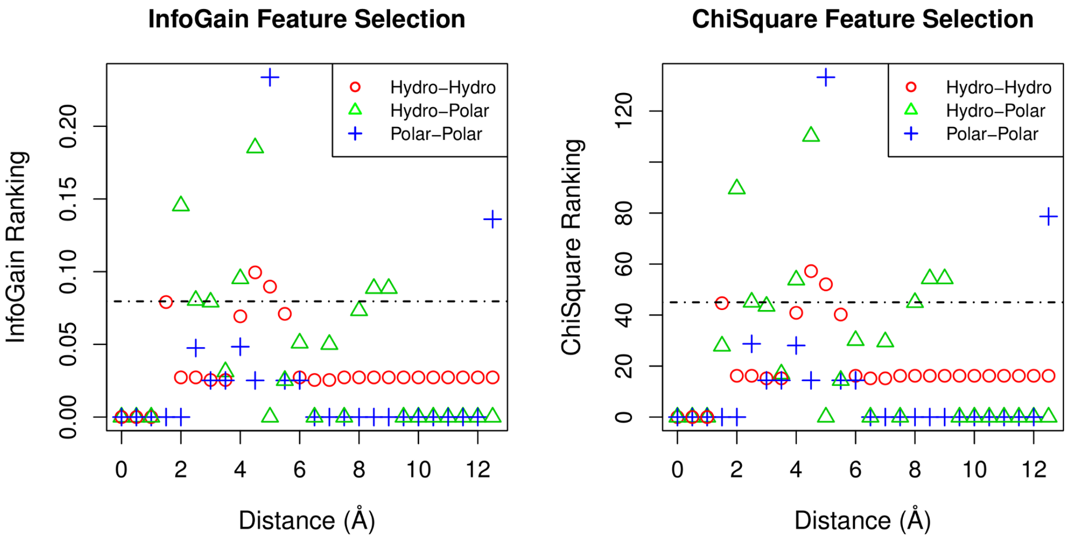

Fig. 6. Feature selection analysis for signatures generated for the ProNIT data set (protein-DNA affinity change upon mutation). The graphs show the attribute ranking according to two different feature selection approaches, which evaluate the discriminative ability of the attributes according to an Information Gain or a Chi-Square statistic (Yang and Pedersen, 1997). The signatures were generated considering two atom classes (hydrophobic and polar) for distances up to 12.5Å. The dashed lines separate the ten best-ranked attributes for each feature selection method. With the exception of two polar-polar features, the most discriminative attributes seem to hydrophobic-polar and hydrophobic-hydrophobic distance patterns.

4 EVALUATION PROTOCOL

4.1 Signature parameter evaluation

In order to identify the best performing configuration for the signatures as well as to study the impact of the different parameters in predictive performance, we developed an evaluation protocol. This protocol was used in regression tasks for stability and affinity change prediction. The main aspects evaluated were:

- *Signature type:* all three signatures were evaluated (mCSM, mCSM-HP and mCSM-ALL);
- *Residue environment cutoff:* distances in the range [5,15]Å, with a step of 1Å, were evaluated. The maximum distance parameter (D_{MAX}) is set to the distance used to define the residue environment;
- *Distance step (D_{STEP}):* distances of 0.5Å, 1.0Å, 1.5Å and 2.0Å were evaluated;
- *Machine learning algorithms:* regression via Gaussian Process and M5P were tested using the Weka Toolkit. No parameter tuning was performed for these algorithms.

For affinity change prediction tasks we also evaluated the performance for a variant of the signature where the distance distributions are further categorized in *intra-* and *inter-chain* (which doubles the size of the signature vector).

4.2 Low-redundancy sets for blind test and cross validation

The different configurations were tested with new blind test sets for protein stability change and protein-protein affinity change prediction tasks, as well as new cross validation schemes. The new blind-tests were defined in two ways:

- *Position level:* all mutations in a position are either in the test or train set exclusively.
- *Protein level:* all mutations in a protein are either in the test or train set exclusively.

The same applies for the definition of the folds in cross validation. All sets were defined randomly. For the new blind tests we attempted to create new sets with roughly the same number of mutations as the original ones. We assembled two data sets (both available at http://structure.bioc.cam.ac.uk/mcsm):

- *SKEMPI S350:* low-redundancy subset (in position level) derived from the BeAtMuSiC data set with 350 mutants, used in protein-protein affinity change prediction tasks.
- *S2648 S351:* low-redundancy subset (in position level) derived from the S2648 data set with 351 mutants, used in protein stability change prediction tasks.

4.3 Signature parameter analysis

Figure 7 shows the parameter evaluation results in low-redundancy cross validation for protein stability change (a), protein-protein (b) and protein-DNA (c) affinity change prediction. The best performing configuration for the BeAtMuSiC data set was the mCSM-ALL signature, with cutoff step of 2Å considering a residue environment of 10Å. Predictive models trained with the Gaussian Process were superior to those trained with M5P.

For the S2648 data set, the best performing configuration was the mCSM-HP signature, with cutoff step of 2Å considering a residue environment of 8Å. Once again, predictive models trained with the Gaussian Process were superior and this algorithm was selected. On the other hand, for the ProNIT data set, the best performing algorithm was the M5P, with mCSM-ALL signatures generated with a cutoff step of 0.5Å and residue environment of 11Å. It is interesting to notice that the cutoff step parameter seems to affect mostly the mCSM-ALL signature.

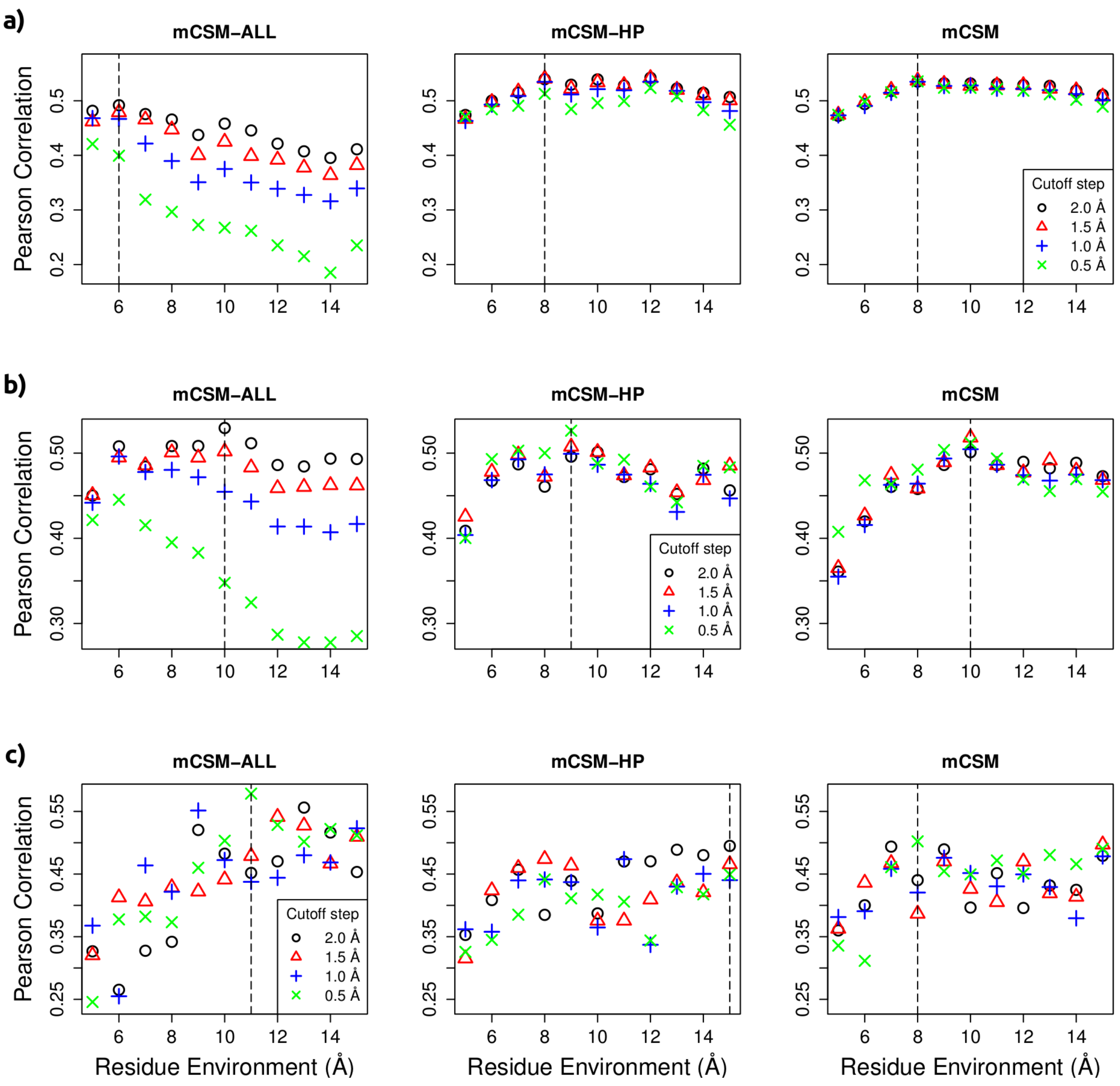

Fig. 7. Signature parameter evaluation. The graphs show the relation between the Pearson correlation coefficient obtained in low redundancy cross-validation and the distance threshold used to define the residue environment. The performances for different cutoff steps are also shown. The best performing configuration is highlighted by a vertical line. (a) Shows the results for 5-fold cross validation (Gaussian Process) for the S2648 data set (protein stability change upon mutation). In (b) are shown the results for protein-protein affinity change prediction regarding the BeAtMuSiC data set, in 10-fold cross validation (Gaussian Process). The results for the ProNIT data set (Protein-DNA affinity change upon mutation 10-fold cross validation, using the M5P algorithm) are shown in graph (c).

4.4 Performance analysis on low-redundancy data set

Tables 8 and 9 summarize the results for the new validation data sets. For the BeAtMuSiC data set (Table 8) mCSM was able to achieve a Pearson's correlation of 0.58 when the signatures are split in Inter/Intra distance patterns. This performance is significantly better than the one achieved by the method BeAtMuSiC (0.40), with a p-value $< 10^{-14}$ (using the Fisher $r - to - z$ transformation, we assessed the significance in the difference of the two correlations by calculating the z value). mCSM also achieved a correlation of 0.56 considering the new blind test, which is compatible with its performance in cross validation.

Table 8. Evaluation of predictive performance of mCSM for the BeAtMuSiC data set in new low-redundancy blind and cross validation schemes. Results are given for data set partitioning in Protein (Prot) and Position (Pos) levels as described in Section 4.2.

Method	Data set	Validation	Pearson's coeff.*	Std. error (Kcal/mol)*
mCSM	BeAtMuSiC	10-fold (Pos)	0.54/0.59	1.60/1.03
mCSM	BeAtMuSiC	10-fold (Prot)	0.53/0.66	1.61/1.05
**mCSM (Inter/Intra)**	**BeAtMuSic**	**10-fold (Pos)**	**0.58/0.71**	**1.55/1.00**
mCSM (Inter/Intra)	BeAtMuSiC	10-fold (Prot)	0.57/0.69	1.55/1.02
**mCSM**	**SKEMPI S350**	**Blind (Pos)**	**0.56**	**1.38**

* When two values are given per column they correspond respectively to the results for whole validation set and after excluding 10% of outliers.

For the task of predicting the stability change upon mutation mCSM was able to achieve 0.54 in terms of Pearson's correlation with a standard error of 1.23 Kcal/mol, as shown in Table 9. For the new low-redundancy blind test, mCSM achieves a correlation of 0.67, in comparison with 0.56 obtained by SDM and 0.73 obtained by PoPMuSiC. It is important to point out that this data set may not be completely blind for PoPMuSiC, since the chosen mutations could have been considered while training its artificial neural network.
It is worth noticing that mCSM and SDM results seem to be complementary. By optimally combining their predictions, it would be possible a theoretical correlation coefficient of 0.82. In future works we plan to evaluate the different scenarios where each method performs best, in order to combine them in a new hybrid approach.

Table 9. Evaluation of predictive performance of mCSM for the S2648 data set in new low-redundancy blind and cross validation schemes. Results are given for data set partitioning in Protein (Prot) and Position (Pos) levels as described in Section 4.2.

Method	Data set	Validation	Pearson's coeff.*	Std. error(Kcal/mol)*
**mCSM**	**S2648**	**5-fold (Pos)**	**0.54/0.69**	**1.23/0.90**
mCSM	S2648	5-fold (Prot)	0.51/0.66	1.26/0.94
mCSM	S2648 S351	Blind (Pos)	0.67	1.19
**mCSM+SDM**	**S2648 S351**	**Blind (Pos)**	**0.82**	**0.91**
PoPMuSiC	S2648 S351	Blind (Pos)	0.73	1.09
SDM	S2648 S351	Blind (Pos)	0.56	0.32

* The two values given per column correspond respectively to the results for whole validation set and after excluding 10% of outliers.

5 METHODS

5.1 Supervised learning

Classification and regression algorithms were used to train models for different tasks related to the impact of mutations in proteins, like predicting stability and affinity change upon mutation and also predicting disease-related mutations. The validation procedure employed for each task and data set can be found in Table 1 of the main text. For all tasks, the Weka Toolkit (Witten and Frank, 2005), developer version 3.6.2 was used.

5.1.1 Regression algorithm

- **Gaussian Process Regression** (Rasmussen and Williams, 2006):

A Gaussian Process $f(x)$, also called Gaussian Random Field, is a collection of random variables that present a joint multivariate normal (Gaussian) distribution. It is specified by a mean function $m(x)$ and a covariance (kernel) function $k(x, x')$:

$$f(x) \sim \mathcal{GP}(m(x), k(x, x'))$$

A Gaussian process can be used as a prior probability distribution over functions in Bayesian inference. Gaussian Process Regression is then a flexible supervised learning approach for regression and non-linear interpolation of data, which also gives uncertainty estimates of the predictions. In this work we used a RBF Kernel as covariance function.

5.1.2 *Classification algorithm*

- **Random Forest** (Breiman, 2001):
  Random Forest is an ensemble method for classification. Learning methods categorised as ensembles use multiple models in order to achieve a better prediction performance than a separate single model. The Random Forest method trains a collection of decision trees (Quinlan, 1986) on random subsets of features available in the input data set and outputs the predicted class by majority voting (i.e., the mode of the classes output by the decision trees). Random Forests have proved to be efficient handling unbalanced and missing data, as well as being efficient in terms of runtime.

5.2 Evaluation metrics

In this section we describe the evaluation metrics used in this work.

5.2.1 *Metrics for regression experiments*

For regression experiments, we used the Pearson's correlation coefficient (ρ) and the standard error (σ).

- **Pearson's correlation coefficient (ρ):** The Pearson's correlation coefficient is a measure of the linear dependence of two variables (in our case, experimental and predicted change in affinity or stability) and is defined as the covariance of the two variables divided by the product of their standard deviations. Its values range from 1 to -1. A correlation of $\rho = 1$ or $\rho = -1$ denotes a perfect linear correlation between the variables, which could be represented as a linear equation with positive and negative slopes, respectively. A correlation of $\rho = 0$ means that there is no linear correlation between the variables.
- **Standard error of regression (σ):** The standard error (σ), also referred to as root mean squared error, is used to quantify differences between real and predicted values by a regression algorithm and can be calculated as follows:

$$\sigma = \sqrt{\sum_{i=1}^{n} \frac{(y_i - \hat{y_i})^2}{n-2}}$$

  where, y_i and $\hat{y_i}$ are, respectively, actual and predicted values, n is the number of observations and $n - 2$ the number of degrees of freedom.

5.2.2 *Metrics for classification experiments*

We define the positive class as the class of mutations that either increase stability in proteins or increase affinity of protein-protein or protein-DNA complexes.

- **True positive (tp)** is an item correctly predicted as belonging to the positive class (e.g., a stabilizing mutation correctly assigned).
- **False positive (fp)** is an item incorrectly predicted as belonging to the positive class (e.g., a stabilizing mutation incorrectly assigned).
- **True negative (tn)** is an item correctly predicted as belonging to the negative class (e.g., a destabilizing mutation correctly assigned).
- **False negative (fn)** is an item incorrectly predicted as belonging to the negative class (e.g., a destabilizing mutation incorrectly assigned).
- **Precision:** is the number of true positives divided by the total number of elements predicted as belonging to the positive class or:

$$Precision = \frac{tp}{tp + fp}$$

- **Recall (Sensitivity):** is the fraction of relevant instances that are retrieved or:

$$Recall = \frac{tp}{tp + fn}$$

- **ROC curves:** Receiver Operating Characteristic curves are derived from traditional confusion matrices. A confusion matrix (Provost and Kohavi, 1998) contains information about actual and predicted classifications done by a classifier and allows performance of classification systems and gives the tn, tp, fp an fn rates. A ROC graph is a plot with the fp rate on the X-axis and the tp rate on the Y-axis. In the ROC space, the point (0,1) indicates a perfect classifier. An interesting feature of these curves is that the area under

curve (AUC) can be used as evaluation metric in many applications. The AUC ranges from 0 to 1 and a random classifier would have an AUC of 0.5.

- **Area under curve (AUC):** is the area under the Receiver Operating Characteristic (ROC) curve and is equal to the probability that a classifier will rank a randomly chosen positive instance higher than a randomly chosen negative one.
- **Mathew's correlation coefficient (MCC):** The Matthews correlation coefficient is a metric of the quality of a classifier that correlates the actual and predicted classes, taking into account all components of a confusion matrix. It is a balanced metric that can be used even for experiments with very different sized classes. MCC values range from -1 to 1, where 1 denotes a perfect classifier, 0 a random classifier and -1 a classifier in total disagreement with actual and predicted classes. From a confusion matrix, the MCC can be calculated as follows:

$$MCC = \frac{tp * tn - fp * fn}{\sqrt{(tp + fp)(tp + fn)(tn + fp)(tn + fn)}}$$

- **Accuracy:** is the fraction of correct results or:

$$Accuracy = \frac{tp + tn}{tp + tn + fp + fn}$$

5.2.3 Training / testing schemes

- **K-fold cross-validation**: Our machine learning approach was tested and evaluated using cross-validation analysis, which is a statistical analysis used to estimate the performance of predictive models, consisting of partitioning the data set into two complementary subsets. The first is the training set used to build the model. The other is the test set used to measure the validity and performance of the model. In *k-fold cross validation*, the data set is partitioned in k equal sized subsets. One subset is retained as test set and the others used as training set. The partition used as test set is varied over k rounds (folds) and the performance metrics are averaged over all the rounds. In our comparative experiments, we used the cross-validation approach described in the original publication (see Table 1 of the main manuscript). In one experiment only we used fixed train/test sets in order to directly compare the performance of our approach with those of others.

REFERENCES

Adzhubei, I. A., Schmidt, S., Peshkin, L., Ramensky, V. E., Gerasimova, A., Bork, P., Kondrashov, A. S., and Sunyaev, S. R. (2010). A method and server for predicting damaging missense mutations. *Nature methods*, **7**(4), 248–249.

Breiman, L. (2001). Random forests. *Machine learning*, **45**(1), 5–32.

Bullock, A. N., Henckel, J., and Fersht, A. R. (2000). Quantitative analysis of residual folding and dna binding in mutant p53 core domain: definition of mutant states for rescue in cancer therapy. *Oncogene*, **19**(10), 1245–1256.

Capriotti, E. and Altman, R. B. (2011). Improving the prediction of disease-related variants using protein three-dimensional structure. *BMC Bioinformatics*, **12**(Suppl 4), S3.

Dehouck, Y., Grosfils, A., Folch, B., Gilis, D., Bogaerts, P., and Rooman, M. (2009). Fast and accurate predictions of protein stability changes upon mutations using statistical potentials and neural networks: PoPMuSiC-2.0. *Bioinformatics*, **25**(19), 2537–2543.

Masso, M. and Vaisman, I. I. (2008). Accurate prediction of stability changes in protein mutants by combining machine learning with structure based computational mutagenesis. *Bioinformatics*, **24**(18), 2002–2009.

Ng, P. C. and Henikoff, S. (2003). Sift: Predicting amino acid changes that affect protein function. *Nucleic Acids Research*, **31**(13), 3812–3814.

Provost, F. and Kohavi, R. (1998). On applied research in machine learning. *Machine Learning*, **30**, 127–132.

Quinlan, J. R. (1986). Induction of decision trees. *Machine learning*, **1**(1), 81–106.

Rasmussen, C. E. and Williams, C. K. (2006). *Gaussian processes for machine learning*, volume 1. MIT Press, Cambridge, MA, USA.

Witten, I. H. and Frank, E. (2005). *Data Mining: Practical Machine Learning Tools and Techniques*. Morgan Kaufmann, second edition.

Worth, C. L., Preissner, R., and Blundell, T. L. (2011). SDM – a server for predicting effects of mutations on protein stability and malfunction. *Nucleic Acids Research*, **39**(suppl 2), W215–W222.

Yang, Y. and Pedersen, J. O. (1997). A comparative study on feature selection in text categorization. In *Proceedings of the 14th International Conference on Machine Learning, ICML*, pages 412–420.
